# Enterococcal colonization of infants in a neonatal intensive care unit: associated predictors, risk factors and seasonal patterns

**DOI:** 10.1186/1471-2334-7-107

**Published:** 2007-09-16

**Authors:** Markus Hufnagel, Cathrin Liese, Claudia Loescher, Mirjam Kunze, Heinrich Proempeler, Reinhard Berner, Marcus Krueger

**Affiliations:** 1 Center for Pediatrics and Adolescent Medicine, University Medical Center Freiburg, Mathildenstr. 1, D-79106 Freiburg, Germany; 2 Department of Gynecology and Obstetrics, University Medical Center Freiburg, Germany

## Abstract

**Background:**

During and shortly after birth, newborn infants are colonized with enterococci. This study analyzes predictors for early enterococcal colonization of infants in a neonatal intensive care unit and describes risk factors associated with multidrugresistant enterococci colonization and its seasonal patterns.

**Methods:**

Over a 12-month period, we performed a prospective epidemiological study in 274 infants admitted to a neonatal intensive care unit. On the first day of life, we compared infants with enterococcal isolates detected in meconium or body cultures to those without. We then tested the association of enterococcal colonization with peripartal predictors/risk factors by using bivariate and multivariate statistical methods.

**Results:**

Twenty-three percent of the infants were colonized with enterococci. The three most common enterococcal species were *E. faecium *(48% of isolates), *E. casseliflavus *(25%) and *E. faecalis *(13%). Fifty-seven percent of the enterococci found were resistant to three of five antibiotic classes, but no vancomycin-resistant isolates were observed. During winter/spring months, the number of enterococci and multidrug-resistant enterococci were higher than in summer/fall months (p = 0.002 and p < 0.0001, respectively). With respect to enterococcal colonization on the first day of life, predictors were prematurity (p = 0.043) and low birth weight (p = 0.011). With respect to colonization with multidrug-resistant enterococci, risk factors were prematurity (p = 0.0006), low birth weight (p < 0.0001) and prepartal antibiotic treatment (p = 0.019). Using logistic regression, we determined that gestational age was the only parameter significantly correlated with multidrug-resistant enterococci colonization. No infection with enterococci or multidrugresistant enterococci in the infants was detected. The outcome of infants with and without enterococcal colonization was the same with respect to death, necrotizing enterocolitis, intracerebral hemorrhage and bronchopulmonary dysplasia.

**Conclusion:**

In neonatal intensive care units, an infant's susceptibility to early colonization with enterococci in general, and his or her risk for colonization with multidrug-resistant enterococci in particular, is increased in preterm newborns, especially during the winter/spring months. The prepartal use of antibiotics with no known activity against enterococci appears to increase the risk for colonization with multidrug-resistant enterococci.

## Background

Although the fetal gastrointestinal tract is considered to be sterile [1-3], colonization of the newborn with microorganisms begins during delivery or within minutes after birth [2,4]. Colonizing microorganisms originate from the vaginal and gastrointestinal flora of the mother, oral ingestion of breast milk and formula milk, or from environmental sources [1-4]. Among the first microorganisms detected in the stool of infants, enterococci are commonly found on the first day of life [1,4]. While enterococci constitute part of the normal intestinal flora of humans (up to 10^8 ^cfu/g stool [5,6]), in smaller numbers, the bacteria are also detectable in the human genital tract and oral cavity. Enterococci are considered facultative pathogens and cause a variety of infections, including urinary tract, intra-abdominal, pelvic, and soft tissue infections, bacteremia and endocarditis [7]. In preterm infants and other immuno-compromised patients, infections with enterococci can be life-threatening [8].

Several factors are known to influence the composition of the microbial flora in infants during and after birth [1,2,4]. Preterm infants and infants delivered via Cesarean section display a delayed intestinal colonization with a smaller species variability and a higher rate of potentially pathogenic microorganisms [1,3,4,9]. Antibiotic treatment both decreases the amount of anaerobic bacteria and increases the number of enterobacteriaceae in an infant's stool [1,4].

In our neonatal intensive care unit, meconium and body cultures are routinely cultured for surveillance purposes. In recent years, we noticed an increase in enterococci – especially aminoglycoside-resistant enterococci – in the meconium of preterm infants admitted to the neonatal intensive care unit shortly after birth. In a retrospective analysis, all enterococci isolated from the meconium over a 12-month period were characterized.

Strikingly, the colonization of preterm infants with enterococci and aminoglycosideresistant enterococci was more prevalent during the winter months than during summer months (unpublished observation). Such an association is well-established for the bacterial colonization of the respiratory tract [10], but has not been known for colonization of infants with enterococci. From the retrospective study, we developed the following two hypotheses: (1) that there is a seasonal influence on the enterococcal colonization of newborn infants in neonatal intensive care units shortly after birth, and (2) that there is a higher risk for the colonization of preterm infants with drug-resistant enterococci. Supporting these hypotheses are two key factors: the generally higher rate of antibiotic use with no known activity against enterococci during the winter/spring season, and the specifically higher rate of antibiotic use by mothers of preterm infants as compared to mothers of term infants.

To confirm our two hypotheses, we conducted a prospective epidemiological study. Over a 12-month period, we monitored colonization of infants with and without enterococci in the meconium and other body cultures shortly after birth and we simultaneously tracked potential risk factors for the acquisition of multidrug-resistant enterococci. Understanding the predictors for newborn infants' early colonization with enterococci is a prerequisite to interpreting potential risk factors associated with multidrug-resistant enterococcal colonization. Colonization with a pathogen that has multidrug resistance poses a risk for the development of an infection [11], which is likely to be difficult to treat with standard antibiotic therapy.

## Methods

### Setting

Freiburg University Medical Center is a 1,800-bed, tertiary care facility in southwestern Germany. The Department of Gynecology and Obstetrics houses an 8-bed neonatal intensive care unit. Approximately 350 patients are admitted to this unit annually.

### Study design

All infants born at the Freiburg University Medical Center between March 1, 2003 and February 28, 2004 and admitted to the neonatal intensive care unit on their first day of life were enrolled in our prospective study. Infants born outside the Freiburg University Medical Center and transferred to the neonatal intensive care unit on their first day of life were excluded. Upon admission to the intensive care unit, specimens were taken from the ear, pharynx and gastric content, and subsequently cultured. The first meconium was also cultured. Clinical data were prospectively collected from the infant (i.e., gestational age, birth weight, sex, symptoms and signs of neonatal infection, bacteremia, antibiotic treatment, symptoms and signs of necrotizing enterocolitis, death) as well as from the mother (i.e., prepartal hospitalization, prepartal antibiotic treatment, premature rupture of membranes, recto-vaginal colonization with enterococci before delivery, mode of delivery). At our hospital, recto-vaginal swabs are routinely taken from pregnant women between week 35 and 37 of their pregnancies in order to screen for colonization with *S. agalactiae*. Prepartum refers to the period immediately preceding delivery of an infant. The study was approved by the Institutional Review Board of the Freiburg University Hospital. Informed consent was obtained from the infant's caretaker.

### Microbiological methods

Meconium and body cultures were processed at the microbiology laboratory of the Center of Pediatrics and Adolescent Medicine Freiburg according to standard methods on solid horse-blood and chocolate agar [12]. After identifying an *Enterococcus *isolate, an API Strep kit was used to determine the enterococcal species. *E. casseliflavus *species was further differentiated from *E. faecalis*/*E. faecium *species using Methyl-a-D-glucopyranosid- and Sulphide-Indole-Motility agar.

Antibiotic resistance was determined using the Kirby-Bauer disc diffusion test. Five different classes of antibiotics were tested (aminopenicillins, carbapenems, aminoglycosides, sulfonamides/folinic acid antagonists and vancomycin). Minimum inhibitory concentrations to aminoglycosides were determined using the E-test [13]. Multidrug resistance was defined by resistance of an enterococcal isolate against three of the five antibiotic classes.

### Statistical analysis

For statistical analysis, Microsoft Excel and the software programs GraphPad Prism V.3 and SPSS 14.0 were used. Results were expressed either as a mean +/- SD, or as a percentage of the total number of isolates or patients. For continuous variables, median values were compared using two sample t-tests for independent variables (i.e., Mann- Whitney test). Differences in proportions were compared using either a Chi-square test or Fisher's exact test, as deemed appropriate. All statistical tests were performed twotailed and considered significant if the p value was <0.05. For multivariate analysis, logistic regression was used.

## Results

### Study population and patient characteristics

During the study period, 297 infants were admitted to the neonatal intensive care unit on their first days of life. Twenty-three infants were excluded from the analysis either because they were born at an outside hospital (n = 16) or because no meconium was cultured (n = 7). Two hundred seventy-four infant/mother pairs were enrolled in this study; 144 infants were male and 130 female. The mean birth weight of the infants was 2.420 g (range 380 to 4.910 g) and the mean gestational age was 35 weeks (range 23 to 44 weeks). One hundred fifty-seven infants were born prematurely, (i.e., before 37 weeks of gestation), and 63 infants were born before 32 weeks of gestation. The study cohort of 274 infants, stratified by three gestational age groups, (i.e., ≥ 37 weeks of gestation, 32 to 36 weeks of gestation and < 32 weeks of gestation), is summarized in Table 1.

**Table 1:** Clinical and peripartal features of infants studied, stratified by gestational age.

**Gestational age**	**n**	**Median (weeks)**	**Range (weeks)**	**Cesarean section**	**Prepartal hospitalization ≥ 3 days**	**Prepartal antibiotics ≥ 1 day**	**PROM^a ^≥ 24 hours**	**Maternal rectovaginal colonization with enterococci**
≥ 37 weeks of gestation	117	39	37–44	62 (53.0%)	4 (3.4%)	5 (4.2%)	2 (1.7%)	33 (28.2%)
32–36 weeks of gestation	94	34	32–36	60 (63.8%)	22 (23.4%)	22 (23.4%)	5 (5.3%)	16 (17.0%)
< 32 weeks of gestation	63	28	23–31	52 (82.5%)	23 (36.5%)	27 (42.9%)	10 (15.9%)	4 (6.3%)

^a^PROM indicates premature rupture of membranes

### Microbiological features

**Figure 1:**
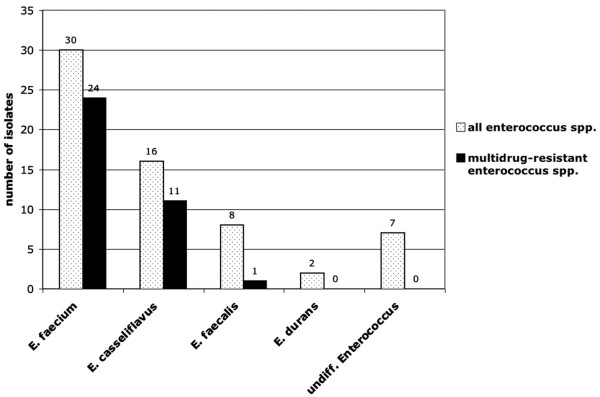
Distribution of different enterococcal species detected in meconium or surveillance cultures of infants (white bars). The black bars represent the number of multidrug-resistant enterococci for each enterococcus species.

In 63 cases (i.e., 23%), enterococci were isolated from either body cultures or from the first meconium. Colonization rates differed according to the three gestational age groups (Table 2). The group with the highest colonization rate was infants < 32 weeks of gestation (i.e., 33%). Of the 63 colonized infants, 53 were isolated from meconium and 10 from body cultures. Differentiation at the species level showed 30 (48% of all enterococcal isolates) *E. faecium*, 16 (31%) *E. casseliflavus*, eight (12%) *E. faecalis*, two (3%) *E. durans*, and seven (11%) undifferentiated enterococcal species (Figure 1). Thirty-six isolates (57% of all enterococcal isolates) were resistant to three of the five antibiotic classes tested and were therefore classified as multidrug-resistant. All of these multidrug-resistant isolates were resistant to aminopenicillins and aminoglycosides. Of the 36 multidrug-resistant enterococci, 35 isolates were resistant to both sulfonamides and carbapenems. In addition to the 36 multidrug-resistant isolates, five other isolates were resistant to aminoglycoside, and all of them displayed low-level aminoglycoside resistance (defined as a minimal inhibitory concentration of gentamicin < 512 μg/ml). None of the enterococci was vancomycin-resistant. Twenty-four (80%) of *E. faecium*, 11 (69%) of *E. casseliflavus*, and one (13%) *E. faecalis *isolate were multidrug-resistant (Figure 1). All multidrug-resistant isolates were detected in meconium.

**Table 2:** Colonization rates of infants with enterococci and multidrug-resistant enterococci in meconium and surveillance cultures stratified by gestational age.

**Gestational age**	**n**	**Colonization with enterococci**	**Colonization with multidrug-resistant enterococci**	**% multidrug-resistant enterococci**
All patients	274	63 (22.9%)	36 (13.1%)	57.1%
≥ 37 weeks of gestation	117	24 (20.5%)	10 (8.5%)	41.7%
32 – 36 weeks of gestation	94	18 (19.1%)	7 (7.4%)	38.9%
< 32 weeks of gestation	63	21 (33.3%)	19 (30.2%)	90.5%

### Seasonal pattern of enterococcal colonization

**Figure 2:**
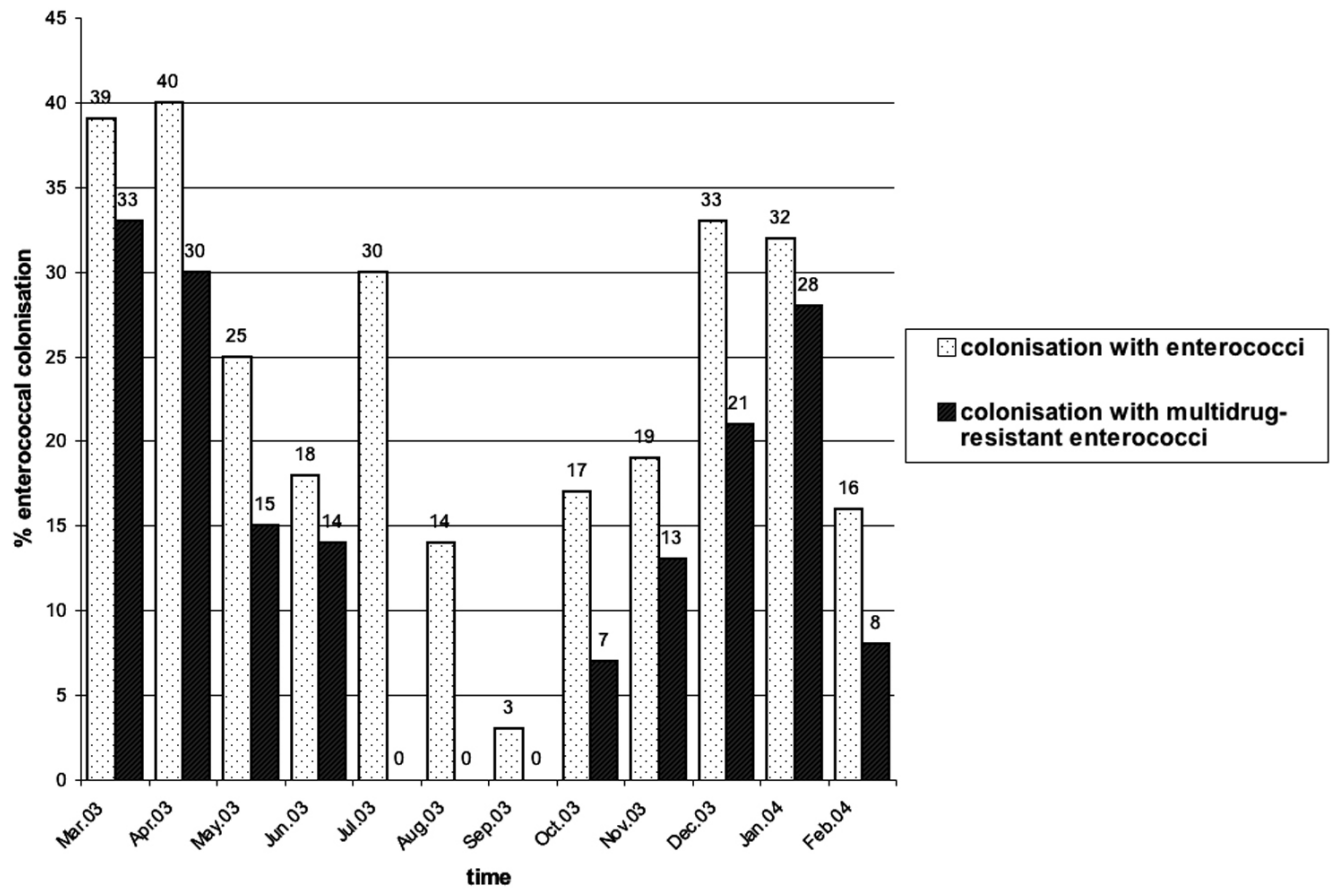
Distribution of isolates of enterococci and multidrug-resistant enterococci (in % of samples taken per month) in meconium and surveillance cultures from colonized infants stratified by month of the year. There is a statistically significant association for a higher colonization rate with enterococci (p = 0.002, Chi-square test) and multidrug-resistant enterococci (p < 0.0001, Chi-square test) during the winter/spring months as compared to summer/fall months.

The 12-month study was divided into two periods: the winter/spring season from December 1^st ^to May 31^st ^and the summer/fall season from June 1^st ^to November 30^th^. A minimum of one enterococcal isolate and a maximum of eight enterococcal isolates were detected per month. A larger number of enterococcal isolates were detected during the winter/spring months as compared to summer/fall months (p = 0.002, Chi-square test, Figure 2). A higher prevalence of enterococcal isolates in the winter/spring season was also noted for multidrug-resistant enterococci, and here with an even higher statistical significance (p < 0.0001, Figure 2).

### Predictors for early colonization with enterococci and multidrug-resistant enterococci

**Figure 3:**
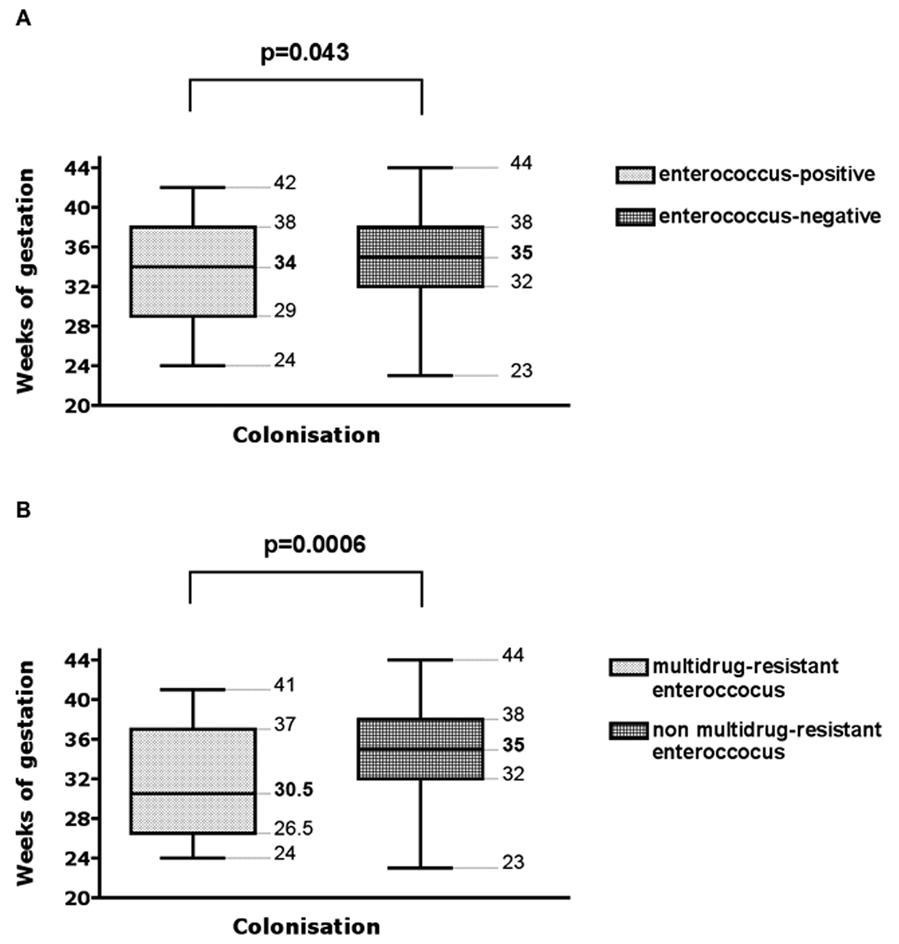
Comparison of gestational age of infants either colonized or not colonized with (A) enterococci and (B) multidrug-resistant enterococci in meconium or surveillance cultures. Boxes extend from the 25th to the 75th percentile, with a line at the median (50th percentile) and whiskers show the highest and the lowest gestational ages. The p values refer to the comparison of the median values using the Mann Whitney test.

**Figure 4:**
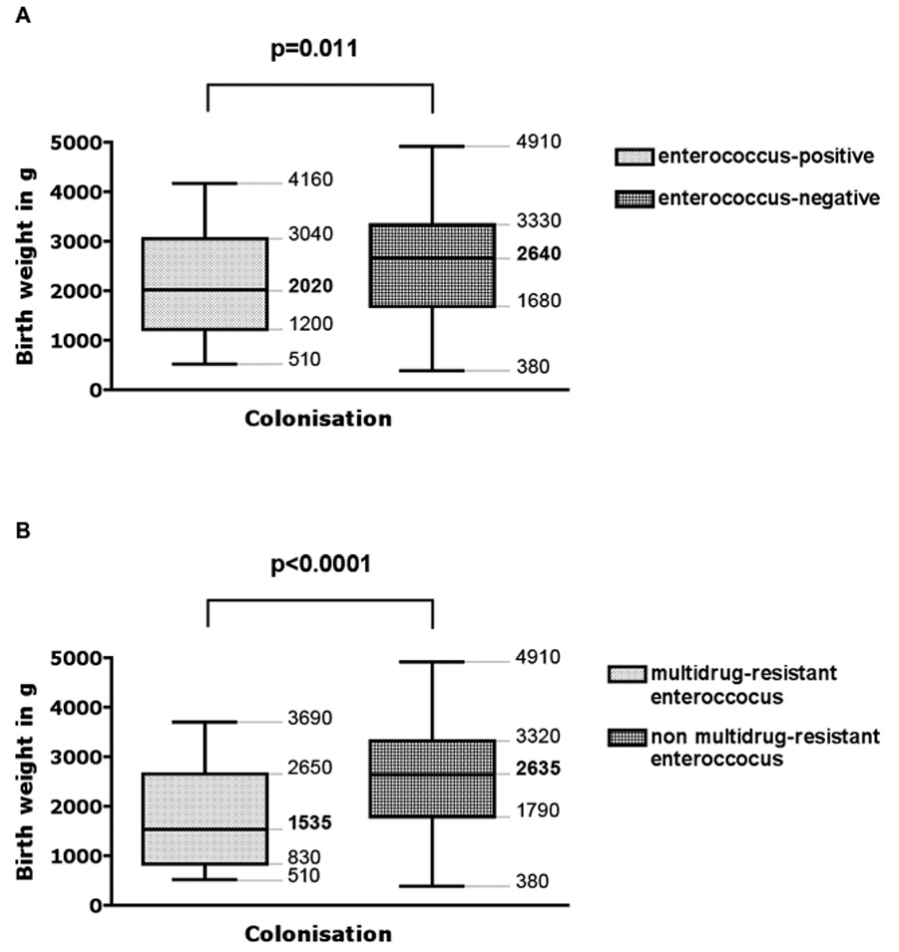
Comparison of birth weights of infants either colonized or not colonized with (A) enterococci and (B) multidrug-resistant enterococci in meconium or surveillance cultures. Boxes extend from the 25th to the 75th percentile, with a line at the median (50th percentile) and whiskers show the highest and the lowest birth weights. The p values refer to the comparison of the median values using the Mann Whitney test.

Different peripartal predictors for colonization with enterococci in infants on their first day of life were analyzed (Table 3). Infants colonized with enterococci had a lower mean gestational age than infants not colonized with enterococci (34 vs. 35 weeks of gestation, p = 0.043, Figure 3A). A similar association was noted between infants colonized with multidrug-resistant enterococci (30.5 vs. 35 weeks of gestation, p = 0.0006, Figure 3B). Infants colonized with enterococci also had a lower mean birth weight than infants not colonized with enterococci (2.020 vs. 2.640 g, p = 0.011, Figure 4A).

**Table 3:** Comparison of peripartal risk factors for colonization with enterococci and multidrugresistant enterococci in meconium and surveillance cultures of infants. For differences in proportions between two groups, the Fisher's exact test was performed.

**1. Gestational age**	**< 32 weeks** (n = 63)	**≥ 32 weeks** (n = 211)	**Relative risk (95% CI)**	**p**
Enteroccous-positive	21 (33.3%)	42 (19.9%)	1.675 (1.076–2.605)	0.0395
^a^MDR-enterococcus-positive	19 (30.2%)	17 (8.1%)	3.743 (2.073–6.759)	<0.0001
**2. Birth weight**	**< 2.500 g** (n = 138)	**≥ 2.500 g** (n = 136)		
Enterococcus-positive	38 (27.5%)	25 (18.4%)	1.498 (0.959–2.340)	0.085
^a^MDR-enterococcus-positive	25 (18.1%)	11 (8.1%)	2.240 (1.148–4.371)	0.019
	**< 1.000 g** (n = 27)	**≥ 1.000 g** (n = 247)		
Enterococcus-positive	11 (40.7%)	52 (21.1%)	1.935 (1.156–3.239)	0.0293
^a^MDR-enterococcus-positive	11 (40.7%)	25 (10.1%)	4.025 (2.237–7.244)	0.0001

**3. Prepartal hospitalization**	**≥ 3 days** (n = 49)	**< 3 days** (n = 224)		
Enterococcus-positive	11 (22.4%)	52 (23.2%)	0.967 (0.546–1.714)	1.000
^a^MDR-enterococcus-positive	7 (14.3%)	29 (12.9%)	1.108 (0.516–2.383)	0.816

**4. Prepartal antibiotics**	**≥ 1 day** (n = 54)	**< 1 day** (n = 216)		
Enterococcus-positive	16 (29.6%)	42 (19.4%)	1.460 (0.893–2.388)	0.146
^a^MDR-enterococcus-positive	12 (22.2%)	21 (9.7%)	2.286 (1.201–4.351)	0.019

**5. Delivery mode**	**Cesarean section** (n = 174)	**Vaginal delivery** (n = 100)		
Enterococcus-positive	42 (24.1%)	21 (21.0%)	1.149 (0.724–1.826)	0.655
^a^MDR-enterococcus-positive	28 (16.1%)	8 (8.0%)	2.011 (0.954–4.243)	0.0641

**6. Maternal rectovaginal colonization**	**Enterococcus-positive** (n = 53)	**Enterococcus-negative** (n = 47)		
Enterococcus-positive	9 (17.0%)	8 (17.0%)	0.998 (0.419–2.374)	1.000
^a^MDR-enterococcus-positive	1 (1.9%)	2 (2.1%)	0.443 (0.042–4.737)	0.600

**7. PROM**^b^	**≥ 24 hours** (n = 17)	**< 24 hours** (n = 248)		
Enterococcus-positive	2 (11.8%)	58 (23.4%)	0.478 (0.128–1.791)	0.376
^a^MDR-enterococcus-positive	2 (11.8%)	32 (12.9%)	0.912 (0.238–3.488)	1.000

^a^MDR indicates multidrug-resistant, ^b^PROM premature rupture of membranes

Colonization with multidrug-resistant enterococci revealed a similar association (1.535 vs. 2.635 g, p < 0.0001, Figure 4B). A 2 × 2 comparison of gestational age groups (< 32 weeks vs. ≥ 32 weeks of gestation) and birth weight groups (< 1.000 g vs. ≥ 1.000 g) confirmed their association with the enterococcal colonization status (Table 3). Prepartal hospitalization, delivery mode, premature rupture of membranes and maternal rectovaginal colonization with enterococci were not associated with the infant's colonization with enterococci or multidrug-resistant enterococci (Table 3). In contrast, however, prepartal antibiotic treatment was associated with colonization of infants with multidrugresistant enterococci (p = 0.019, Table 3) although not with enterococcal colonization (p = 0.146, Table 3). The following antibiotics were used for mothers of infants subsequently colonized with enterococci: cefuroxime (n = 17), ampicillin (n = 2), erythromycin (n = 1), ceftriaxone (n = 1), clindamycin (n = 1) and an unknown antibiotic (n = 1). Standard route of antibiotic administration was parenteral injection. Logistic regression determined that gestational age was the only parameter significantly correlated with colonization with multidrug-resistant enterococci (Table 4). Further modeling was not deemed appropriate because of the strong correlation between gestational age and the parameters of birth weight and prepartal antibiotic treatment.

**Table 4:** Logistic regression of risk factors associated for colonization with enterococci and multidrug-resistant enterococci (inclusion model)

	**Colonization with**			
	**Enterococci**		**Multidrug-resistant enterococci**	
	OR (95% CI)	P	OR (95% CI)	P

**Gestational age < 32 weeks**	2.116 (0.896–4.990)	0.087	5.307 (1.723–16.347)	0.004
**Birth weight < 2500 g**	1.150 (0.511–2.582)	0.736	0.861 (0.260–2.845)	0.806
**Prepartal hospitalization ≥ 3 days**	1.665 (0.680–4.078)	0.265	1.878 (0.625–5.638)	0.261
**Prepartal antibiotics ≥ 1 day**	0.556 (0.234–1.322)	0.184	0.461 (0.160–1.328)	0.151
**Cesarian section**	1.182 (0.603–2.315)	0.625	1.908 (0.729–4.995)	0.188
**PROM**^a^	4.866 (0.928–25.488)	0.061	3.705 (0.653–21.043)	0.139

^a^PROM indicates premature rupture of membranes

### Outcome

None of the infants colonized with enterococci shortly after birth developed an infection with enterococci or enterococcal bacteremia. The rate of infants suffering from necrotizing enterocolitis (i.e., 4.8% of the infants colonized with enterococci vs. 1.9% of the infants not colonized with enterococci, p = 0.083) was similar in both groups. Mortality rates during hospitalization also did not differ between the two groups (i.e., 3.2% vs. 4.3%, p = 1.000). In addition, the rate of infants who developed an intracerebral hemorrhage or bronchopulmonary dysplasia was similar between colonized and non- colonized infants (data not shown).

## Discussion

The results of our study demonstrate for the first time that newborn infants can be colonized with multidrug-resistant enterococci from the first day of life. We determined a possible risk factor to be prepartal antibiotic treatment. Knowledge of the possibility of early colonization with multidrug-resistant enterococci is particularly important in cases where such an infant develops an infection and requires appropriate antibiotic treatment.

Enterococci are among the first bacteria to colonize the neonatal gastrointestinal tract. They do so within days of birth [2-4,14]. Although a primary source for the colonizing bacteria is the oral ingestion of breast milk [15,16], bacteria can also be derived from the vaginal and gastrointestinal flora of the mother during the birth passage [14]. Older medical literature suggests the fetal gastrointestinal tract to be sterile [1-3]. More recent studies have shown, however, that infants may be colonized with enterococci and other gram-positive bacteria without having had direct contact with the maternal flora during delivery and before having been fed [14,17,18]. Jimenez at al. detected enterococci by culture after enrichment in nine of 20 cord blood samples from infants delivered via Cesarean section [14]. In experiments with pregnant mice orally fed with a genetically labeled *E. faecium*, the same enterococcal strain was found in the amnion fluid by polymerase chain reaction (PCR) before the mice gave birth [14]. A potential explanation for this finding is the transfer of enterococci ingested by dendritic cells from the gastrointestinal lumen through the gut epithelium into the circulation [19].

No study about colonization of infants' gastrointestinal tracts after birth has systematically differentiated among different enterococcal species involved in this process and/or the influence of peripartal antibiotic treatment. The aforementioned study by Jimenez et al. detected *E. faecium *in cord blood of infants after Cesarean section [14]. By day ten after birth, infants with extremely low birth weights were colonized more often with *E. faecalis *than with *E. faecium *strains [9]. Our cohort was colonized primarily with *E. faecium *(48% of all enterococcal strains), followed by *E. casseliflavus *(31%) and then *E. faecalis *(12%, Figure 1).

This distribution pattern appears to contrast with the enterococcal species distribution causing infection in humans. The most common enterococcal pathogen is *E. faecalis*, which is responsible for 80–90% of enterococcal infection, followed by *E. faecium*, which is responsible in 5–10% of cases [20]. Motile enterococci like *E. casseliflavus *and *E. gallinarum *are responsible for ≈1% of all enterococcal infections [20,21]. Immunocompromised patients have an especially increased risk of contracting infections with motile enterococci [21], which are part of the normal human gastrointestinal flora [21]. In contrast to infection rates, colonization rates of healthy humans with motile enterococci are reported to be higher (5.7% to 12.1%) [21]. A possible explanation for the high prevalence of *E. faecium *strains in our cohort is the high rate of multidrug resistance. This may give *E. faecium *strains a selective advantage for colonization, particularly in the presence of antibiotic treatment. Eighty percent of *E. faecium *and 69% of *E. casseliflavus *isolates in our cohort were resistant to three of the five antibiotic classes tested, whereas only 12% of *E. faecalis *strains were multidrug-resistant.

An association between the rate of enterococcal colonization and prematurity has been previously reported in the literature [22]. In a retrospective case-control study, Miedema et al. analyzed risk factors for enterococcal colonization in 579 infants in a neonatal intensive care unit [22]. Twenty percent of their infants were colonized with enterococci (without further species differentiation) either upon admission to the unit or else during their hospitalization. In a multiple regression analysis, prematurity, days of hospitalization, deep venous line in place, and antibiotics other than amoxicillin were independent risk factors for enterococcal colonization [22]. Miedema's study cohort did not differentiate between antibiotic-sensitive and drug-resistant enterococci. In our cohort, both prematurity and low birth weight were predictors for early colonization with enterococci (Figure 2 and 3). This association was independent of maternal antibiotic use during pregnancy (Table 3). Two other studies have shown that maternal antibiotic treatment does not have an influence on the bacterial colonization in infants after the tenth day of life [9,23]. Using culture methods, Gewolb et al. investigated the stool flora of 29 extremely low birth weight infants on day 10, 20 and 30 of life. The bacterial flora did not differ between infants with or without maternal antibiotic treatment around the time of birth [9]. Using real-time PCR, Penders et al. studied the bacterial flora in stool of 1032 infants at one month of age. The colonization pattern they detected was independent from maternal antibiotic treatment [23]. In our cohort, however, maternal antibiotic treatment proved to be a risk factor for infants' colonization with multidrugresistant enterococci shortly after birth (Table 3). Antibiotics used in our cohort were primarily cephalosporins (17 of 23 cases with prepartal antibiotic treatment and subsequent enterococcal colonization in infants). Because of the high resistance rates of *E. coli *to aminopenicillins, cephalosporins are the antibiotics standardly used by our hospital for women experiencing preterm labor. By contrast, only one pregnant woman who was treated with an aminopenicillin gave birth to an infant colonized with enterococci. Enterococci show an intrinsic resistance against cephalosporins. The use of cephalosporins may therefore be responsible for the selective advantage of enterococci in our cohort. It is unclear to us why the results of our study revealed only an association between the prepartal use of antibiotics (mainly cephalosporins) and the colonization with multidrug-resistant enterococci, but not enterococci in general – this being the case despite the fact that all enterococci are resistant to cephalosporins. The association between the use of cephalosporins and subsequent colonization with ampicillin-resistant enterococci has been previously reported in a cohort of adult patients [24]. An explanation for this phenomenon was not provided by the authors of this study.

The most striking finding of our study is the higher prevalence of early colonization with enterococci and multidrug-resistant enterococci in the winter/spring months as compared to summer/fall months (Figure 1). The same seasonal pattern was observed at our hospital the year before this prospective study was performed (data not shown). A similar seasonal pattern has been reported only in association with colonization with vancomycin-resistant *E. faecium *(VREF) [25]. Bischoff et al. described the molecular epidemiology of 413 vancomycin-resistant isolates from a large urban hospital in Richmond, Virginia (USA), over a five-year period. They noted higher rates of VREF isolation during the winter/spring months. An explanation for this finding was not directly presented by their study. With *S. pneumoniae*, it is well documented that the prevalence of pneumococci is higher in winter [10,26,27]. Another recent study also noted a higher rate of penicillin-resistant pneumococci in acute otitis media cases during winter months [28]. The authors speculate that the association may be explained by the higher rate of antimicrobial use in the population during winter because of the frequency of respiratory tract infection at that time of the year.

The same increased seasonal use of antibiotics in the population may explain the higher rate of multidrug-resistant enterococci that we detected in our cohort during the winter and spring months. Winter and spring are the periods with the highest rates of respiratory tract infections – at least in the northern hemisphere. In Germany, a surveillance system for respiratory tract infections in children has found that for the season 2003/2004, the rate of respiratory tract infections began in early December 2003 and ended in May 2004 [29]. Outpatient antibiotic use in the European population, documented on a quarterly rather than monthly basis, shows a strongly correlated trend: the highest rates of outpatient antibiotic use in this population are to be found in the first and fourth quarter of the year [30]. Although the combined data from our retrospective and prospective studies suggest a strong link between an increased rate of enterococcal colonization and the winter/spring period, a causal relationship between time of the year and an increased rate of antibiotic use in the population cannot yet be conclusively drawn. Further studies will be needed to confirm this connection.

## Conclusion

The current study has demonstrated that colonization of newborn infants with enterococci and drug-resistant enterococci is increased in preterm infants, and colonization with a multidrug-resistant strain may occur around or shortly after birth. Additionally, we have observed that colonization with multidrug-resistant enterococci tends to be more prevalent in winter and spring months. This increased risk is independent both of the mother's length of prepartal hospitalization and of the mode of delivery. Prepartal use of antibiotics with no known activity against enterococci appears to increase the risk of early colonization with drug-resistant enterococci, although notably not with enterococci overall. None of the infants colonized with enterococci developed an infection with enterococci or enterococcal bacteremia. In our cohort, early colonization of infants with enterococci or drug-resistant enterococci had no influence on the survival of the infants or on the rates of necrotizing enterocolitis.

## Competing interests

The author(s) declare that they have no competing interests.

## Authors' contributions

MH participated in the statistical analysis, in the interpretation of the data and in the drafting of the manuscript. CaL participated in the statistical analysis, in the interpretation of the data and in the drafting of the manuscript. CIL and MiK conducted the data collection. HP and RB contributed to the study design and to the interpretation of the data. MaK designed the study, participated in the statistical analysis, interpretation of the data and in the editing of the manuscript. All authors read and approved the final version of the manuscript.

## Pre-publication history

The pre-publication history for this paper can be accessed here:



## References

[B1] Fanaro S, Chierici R, Guerrini P, Vigi V (2003). Intestinal microflora in early infancy: composition and development. Acta Paediatr Suppl.

[B2] Mackie RI, Sghir A, Gaskin HR (1999). Developmental microbial ecology of the neonatal gastrointestinal tract. Am J Clin Nutr.

[B3] Schwiertz A, Gruhl B, Löbnitz M, Michel P, Radke P, Blaut M (2003). Development of the intestinal bacterial composition in hospitalized preterm infants in comparison with breast-fed, full-term infants. Pediatr Res.

[B4] Orrhage K, Nord CE (1999). Factors controlling the bacterial colonization of the intestine in breastfed infants. Acta Paediatr Suppl.

[B5] Huycke MM, Sahm DF, Gilmore MS (1998). Multiple-drug resistant enterococci: the nature of the problem and an agenda for the future. Emerg Infect Dis.

[B6] Noble CJ (1978). Carriage of group D streptococci in the human bowel. J Clin Pathol.

[B7] Murray BE (2000). Vancomycin-resistant enterococcal infections. N Engl J Med.

[B8] Maki DG, Egger WA (1988). Enterococcal bacteremia: clinical features, risk of endocarditis, and management. Medicine.

[B9] Gewolb IH, Schwalbe RS, Taciak VL, Harrison TS, Panigrahi P (1999). Stool microflora in extremely low birthweight infants. Arch Dis Child Fetal Neonatal Ed.

[B10] Hendley JO, Hayden FG, Winther B (2005). Weekly point prevalence of *Streptococcus pneumoniae, Hemophilus influenzae *and *Moraxella catarrhalis *in the upper airways of normal young children: effect of respiratory illness and season. APMIS.

[B11] Huang YC, Chou YH, Su LH, Lien RL, Lin TY (2006). Methicillin-resistant *Staphylococcus aureus *colonization and its association with infection among infants hospitalized in neonatal intensive care units. Pediatr.

[B12] Facklam R, Collins MD (1989). Identification of *Enterococcus *species isolated from human infections by a conventional test scheme. J Clin Microbiol.

[B13] National Committee for Clinical Laboratory Standards (1999). Performance standards for antimicrobial susceptibility testing (supplement). Villanova, PA.

[B14] Jimenez E, Fernandez L, Marin ML, Martin R, Odriozola JM, Nueno-Palop C, Narbad A, Olivares M, Xaus J, Rodriguez JM (2005). Isolation of commensal bacteria from umbilical cord blood of healthy neonates born by cesarean section. Curr Microbiol.

[B15] Martin R, Langa S, Reviriego C, Jimenez E, Marin ML, Xaus J, Fernandez L, Rodriguez JM (2003). Human milk is a source of lactic acid bacteria for the infant gut. J Pediatr.

[B16] Heikkilä MP, Saris PEJ (2003). Inhibition of *Staphylococcus aureus *by the commensal bacteria of human milk. J Appl Microbiol.

[B17] Hitti J, Riley DE, Krohn MA, Hillier SL, Agnew KJ, Krieger JN, Eschenbach DA (1997). Broad-spectrum bacterial ribosomal RNA polymerase chain reaction for detecting amniotic fluid infection among women in preterm labor. Clin Infect Dis.

[B18] Bearfield C, Davenport ES, Sivapathasundaram V, Allaker RP (2002). Possible association between amniotic fluid micro-organism infection and microflora of the mouth. Br J Obstet Gynaecol.

[B19] Rescigno M, Urbano M, Valzasina B, Francolini M, Rotta B, Bonasio R, Granucci F, Kraehenbuhl JP, Ricciardi-Castagnoli P (2001). Dendritic cells express tight junction proteins and penetrate gut epithelial monolayers to sample bacteria. Nat Immunol.

[B20] Murray BE (1990). The life and times of the enterococcus. Clin Microbiol Rev.

[B21] Reid KC, Cockerill FR, Patel R (2001). Clinical and epidemiological features of *Enterococcus casseliflavus/flavescens *and *Enterococcus gallinarum *bacteremia: a report of 20 cases. Clin Infect Dis.

[B22] Miedema CJ, Kerkhof M, Arends JP, Bergman KA, Kimpen JLL (2000). Risk factors for colonization with enterococci in a neonatal intensive car unit. Clin Microbiol Infect.

[B23] Penders J, Thijs C, Vink C, Stelma FF, Snijders B, Kummeling I, van den Brandt PA, Stobberingh EE (2006). Factors influencing the composition of the intestinal microbiota in early infancy. Pediatr.

[B24] Weinstein JW, Roe M, Towns M, Sanders L, Thorpe JJ, Corey GR, Saxton DJ (1996). Resistant enterococci: a prospective study of prevalence, incidence, and factors associated with colonization in a university hospital. Infect Control Hosp Epidemiol.

[B25] Bischoff WE, Reynolds TM, Hall GO, Wenzel RP, Edmond MB (1999). Molecular epidemiology of vancomycin-resistant *Enterococcus faecium *in a large urban hospital over a 5-year period. J Clin Microbiol.

[B26] Prellner K, Christensen P, Hovelius B, Rosen C (1984). Nasopharyngeal carriage of bacteria in otitis-prone and non-otitis-prone children in day-care centers. Acta Otolaryngologica.

[B27] Syrjänen RK, Kilpi TM, Kaijalainen TH, Herva EE, Takal AK (2001). Nasopharyngeal carriage of *Streptococcus pneumoniae *in Finnish children younger than 2 years old. J Infect Dis.

[B28] Hoberman A, Paradise JL, Greenberg DP, Wald ER, Kearney DH, Colborn DK (2005). Penicillin susceptibility of pneumococcal isolates causing acute otitis media in children. Pediatr Infect Dis J.

[B29] Pediatric Infectious Diseases Network on Acute Respiratory Tract Infections. http://www.pid-ari.net/RL_WebWarn/Saisonberichte.htm.

[B30] Ferech M, Coenen S, Malhotra-Kumar S, Dvorakova K, Hendrckx E, Suetens C, Goosens H, ESAC Project Group (2006). European surveillance of antimicrobial consumption (ESAC): outpatient antibiotic use in Europe. J Antimicrob Chemother.

